# Antibacterial Activity of Alanine-Derived Gemini Quaternary Ammonium Compounds

**DOI:** 10.1007/s11743-015-1778-3

**Published:** 2015-12-30

**Authors:** Agata Piecuch, Ewa Obłąk, Katarzyna Guz-Regner

**Affiliations:** Institute of Genetics and Microbiology, University of Wrocław, Przybyszewskiego 63/77, 51-148 Wrocław, Poland

**Keywords:** Gemini quaternary ammonium compounds, Biofilm, Adhesion, *Pseudomonas aeruginosa*, *Staphylococcus epidermidis*

## Abstract

The antibacterial activity of alanine-derived gemini quaternary ammonium salts (chlorides and bromides) with various spacer and alkyl chain lengths was investigated. The studied compounds exhibited a strong bactericidal effect, especially bromides with 10 and 12 carbon alkyl chains and 3 carbon spacer groups (TMPAL-10 Br and TMPAL-12 Br), with a short contact time. Both salts dislodged biofilms of *Pseudomonas aeruginosa* and *Staphylococcus epidermidis*, and were lethal to adherent cells of *S. epidermidis.* Bromide with 2 carbon spacer groups and 12 carbon alkyl chains (TMEAL-12 Br) effectively reduced microbial adhesion by coating polystyrene and silicone surfaces. The results obtained suggest that, after further studies, gemini QAS might be considered as antimicrobial agents in medicine or industry.

## Introduction

Quaternary ammonium salts (QAS) are commonly used in medicine and industry. These cationic surfactants are applied as preservatives, biocides, disinfectants and muscle relaxants [1, 2]. Gemini quaternary ammonium salts (gemini QAS) are built of two monomeric QAS molecules linked by a spacer. Their good surface activity is due to the presence of two hydrophilic head groups and two hydrophobic alkyl chains. Gemini QAS have much lower CMC (critical micelle concentrations) in comparison to monomeric surfactants [3]. Gemini QAS surfactants are able to form bilayer aggregates, like liposomes, and are extensively studied as potential non-viral gene delivery systems or drug carriers [4–6]. The activity of gemini QAS against microorganisms is generally stronger in comparison to the corresponding monomeric compounds and depends on the structure of the gemini molecule [7].

Previous research regarding gemini QAS with betaine ester type arrangements showed that low concentrations of these compounds inhibited bacterial and fungal growth. This activity depended on the alkyl chain lengths, spacer structure and the counterion, with the greatest growth reduction being exhibited by *N*,*N*′-bis[2-dodecyloxy-2-oxoethyl]-*N*,*N*,*N*′,*N*′-tetramethylethane-1,2-diammonium dichloride. The betaine ester gemini surfactants strongly affected bacterial and fungal biofilms (i.e., multicellular communities surrounded by extracellular polymeric substances). Moreover, these compounds inhibited cell adhesion and prevented biofilm formation by coating the surface. Strong anti-biofilm activity was observed especially against *Pseudomonas aeruginosa* and *Staphylococcus epidermidis* biofilms [8, 9]. These two species are common contaminants of hospital environment and a frequent cause of nosocomial infections. *P. aeruginosa* is especially dangerous for cystic fibrosis (CF) patients, who are vulnerable to lung infections. The formation of *P. aeruginosa* biofilms is promoted by many determinants (e.g., fimbriae, proteins, eDNA). Biofilm cells are surrounded by a polysaccharide matrix that plays an important role in biofilm maintenance and resistance to antibiotics [10, 11].

*S. epidermidis* colonizes human skin and mucosal membranes. It is also a common contamination of medical devices (e.g., catheters) and a cause of bacteremia, mainly in immunocompromised patients and neonates. The virulence of *S. epidermidis* is often associated with the ability of this species to form biofilms on polymeric surfaces. Intercellular adhesion and biofilm accumulation is mediated by several factors, such as PIA (polysaccharide intercellular adhesion), Aap (accumulation associated protein) or Embp (extracellular matrix binding protein) [12, 13].

Biofilms are extremely hard to eliminate. Adherent cells usually exhibit antibiotic tolerance due to the altered metabolism and components of the biofilm matrix. Polymeric extracellular matrix, enriched in eDNA and proteins, makes the biofilm structure robust and resistant to eradication, and thus there is a need for new compounds with good anti-biofilm properties [14, 15].

To investigate whether the antimicrobial activity depends on the head group structure, a series of gemini QAS (with different alkyl chain and spacer lengths) that mimic alanine was synthesized [16]. In the present study we investigate their biological activity against Gram-positive and Gram-negative bacteria as well as anti-adhesive and biofilm dislodging capacities.

## Experimental Methods

### Chemicals

**Fig. 1:**
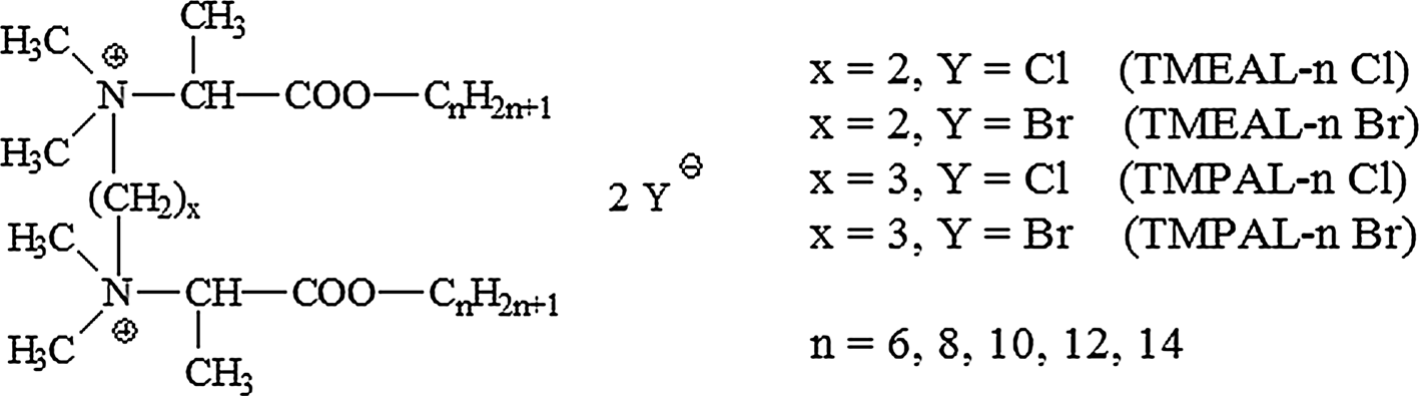
Structure of tested gemini quaternary ammonium, derivatives of *N*,*N*,*N*′,*N*′-tetramethylethylenediamine: TMEAL-*n* Br (*N*,*N*-bis(1-(*n*-alkyloxy)-1-oxopronan-2-yl)-*N*,*N*,*N*′,*N*′-tetramethylethane-1,2-diammonium dibromide); TMEAL-*n* Cl (*N*,*N*′-bis(1-(*n*-alkyloxy)-1-oxopronan-2-yl)-*N*,*N*,*N*′,*N*′-tetramethylethane-1,2-diammonium dichloride) or *N*,*N*,*N*′,*N*′-tetramethyl-1,3-propanediamine: TMPAL-n Br *N*,*N*′-bis(1-(*n*-alkyloxy)-1-oxopronan-2-yl)-*N*,*N*,*N*′,*N*′-tetramethylpropane-1,3-diammonium dibromide; TMPAL-n Cl (*N*,*N*′-bis(1-(*n*-alkyloxy)-1-oxopronan-2-yl)-*N*,*N*,*N*′,*N*′-tetramethylpropane-1,3-diammonium dichloride)

The structure of the alanine-derived gemini quaternary ammonium salts (QAS), synthesized at the Department of Chemistry, Technical University of Wroclaw, Poland, as described previously [16] is shown in Fig. 1.

### Strains

In the present study we used the following clinical strains: *Staphylococcus epidermidis* B374 MRSE, *Enterococcus faecalis* 30VRE (resistant to vancomycin) from Wroclaw Medical University as well as reference strains: *Staphylococcus aureus* ATCC 6538, *Escherichia coli* ATCC 11229 and *Pseudomonas aeruginosa* PAO1 from Institute of Genetics and Microbiology, University of Wrocław collection.

### Evaluation of Minimal Inhibitory Concentration (MIC) and Minimal Bactericidal Concentration (MBC)

Minimal inhibitory concentrations of the studied gemini QAS (at a concentration range of 1–800 µM) against bacterial strains were determined using the broth dilution method recommended by NCCLS, M7-A5 [17]. Strains were incubated with or without (growth control) gemini QAS compounds for 24 h at 37 °C. Each sample was run in duplicate. The MIC values were determined spectrophotometrically and expressed as the concentration of the gemini surfactant that completely inhibited bacterial growth. Optical density of each well was measured at λ_550_ using an 96-well microplate reader (Asys Hitachi 340, Driver Version: 4.02, Biogenet, Poland). The experiment was repeated three times.

To determine the minimal bactericidal concentration (MBC) 100 µl of bacterial suspension incubated with gemini QAS at the MIC, 2 × MIC and 4 × MIC was transferred to Luria Broth (1 % tryptone, 1 % yeast extract, 0.5 % NaCl) plate. MBC were expressed as the concentration of the gemini surfactant that reduced the number of colony forming units (cfu) by 99.9 % after 24 h of incubation at 37 °C, as described elsewhere [18].

### Short-Time Killing Assay

Short-time killing assay was undertaken using *S. epidermidis* B374 strain to determine bactericidal activity of gemini QAS. Bacterial cultures were suspended in physiologic salt solution overnight and turbidity was adjusted to the 0.5 standards of the McFarland scale. Suspensions were then diluted to obtain 10^4^ cells/ml in LB medium. Gemini surfactants were added to the bacterial suspensions to obtain a final concentration equal to the MBC. Cells were incubated at 37 °C with constant agitation (250 rpm). At each time point (0, 5, 15, 30, 60 and 120 min) samples (10 µl) were transferred onto LB agar plates. Plates were incubated at 37 °C for 24 h and colonies were counted.

### Adhesion Inhibition

The reduction of bacterial adhesion to polystyrene and silicone surfaces was determined according to Cremet *et al*. and Silva, respectively [19, 20]. Briefly, a 96-well polystyrene microtiter plate or 2 cm fragments of silicone catheters were incubated with various concentrations of gemini QAS for 2 h at 37 °C and washed with distilled water. A sample (100 µl) of *P. aeruginosa* PAO1 or *S. epidermidis* suspended in LB (OD 0.6) was added and the surfaces were incubated for an additional 4 h. After 5-min staining with crystal violet, absorbance was measured at λ_590_ using an Asys Hitachi 340 instrument (Biogenet, Poland).

### Biofilm Viability

*P. aeruginosa* and *S. epidermidis* biofilm viability were tested with a FilmTracer LIVE/DEAD BacLight Biofilm Viability Kit (Invitrogen) according to Obłąk [8]. Biofilms were grown on glass chamber slides in LB medium for 24 h, at 37 °C and washed with distilled water. Gemini surfactants: TMPAL-10 Br, TMPAL-12 Br and TMEAL-12 Br were applied at a concentration of 120 µM and biofilms were incubated for 2 h. The compounds were removed and biofilms were stained with LIVE/DEAD fluorescent dye. For microscopic observations an Olympus BX51 fluorescence microscope was used.

### Statistical Analysis

To estimate the significance of the impact of gemini QAS on bacterial growth and adhesion, the statistical analysis tests were performed using *Statistica 12*. All experiments were repeated at least three times and the significance was stated at a *p* value <0.05.

## Results

### Minimal Inhibitory Concentration (MIC)

The evaluation of minimal inhibitory concentrations allowed us to determine the activity of gemini quaternary ammonium salts with various lengths of alkyl chains (C6–C14) and spacer ((CH_2_)_2_ or (CH_2_)_3_) against Gram-positive (*S. epidermidis*, *S. aureus* and *E. faecalis*) and Gram-negative bacteria (*P. aeruginosa*).

It was shown that the molecular structure of the surfactant had an influence on its antibacterial activity—generally the compounds with 12 carbon atoms exhibited stronger antibacterial activity (*p* = 0.0005). Similarly, gemini QAS with longer spacers (three methylene groups) were more effective in comparison to compounds with two methylene groups within the spacer (*p* = 0.0002) (Table 1).

**Table 1:** Minimal inhibitory concentrations of alanine-derived gemini quaternary ammonium salts

Compound	Minimal inhibitory concentrations (MIC) of tested compounds [μM]				
	*E. faecalis* 30VRE	*S. epidermidis* B374	*S. aureus* ATCC 6538	*E. coli* ATCC 11229	*P. aeruginosa* PAO1
TMEAL-14 Br	>800	>800	>800	300	> 800
TMEAL-12 Cl	800	300	>800	>800	>800
TMEAL-12 Br	80	40	40	200	>800
TMEAL-10 Br	160	160	300	>800	>800
TMEAL-8 Br	>800	>800	>800	>800	>800
TMEAL-6 Br	>800	>800	>800	>800	>800
TMPAL-12 Cl	160	80	80	160	>800
TMPAL-12 Br	20	10	20	40	375
TMPAL-10 Br	10	5	5	10	20

Comparison between the antibacterial effect against Gram-positive and Gram-negative bacteria showed that the impact of gemini QAS on bacterial growth depended on the strain. Generally, two tested strains of staphylococci were rather sensitive to gemini surfactants, whereas vancomycin-resistant *E. faecalis* and *P. aeruginosa* PAO1 exhibited higher tolerance (*p* < 0.04).

It was also shown that there is a correlation between the counterion of a gemini QAS and its antibacterial activity, since the bromides had a stronger effect on bacterial growth than the chlorides (*p* = 0.038).

The strongest bactericidal activity against both Gram-positive and Gram-negative bacteria was exhibited by surfactants with longer spacers and C10–C12 alkyl chains—TMPAL-10 Br and TMPAL-12 Br (MBC of 20–80 µM). Low MIC values against Gram-positive strains were also observed for the compound with a shorter spacer and 12 carbon atoms (TMEAL-12 Br), but the minimal bactericidal concentrations were much higher, indicating a rather bacteriostatic effect of this compound (Tables 1, 2).

**Table 2:** Minimal bactericidal concentrations of alanine-derived gemini quaternary ammonium salts

Compound	Minimal bactericidal concentrations (MBC) [µM] of tested compounds				
	*E. faecalis* 30VRE	*S. epidermidis* B374	*S. aureus* ATCC 6538	*E. coli* ATCC 11229	*P. aeruginosa* PAO1
TMEAL-14 Br	>800	>800	>800	480	>800
TMEAL-12 Cl	>800	800	>800	>800	>800
TMEAL-12 Br	300	300	80	200	>800
TMEAL-10 Br	160	160	800	>800	>800
TMEAL-8 Br	>800	>800	>800	>800	>800
TMEAL-6 Br	>800	>800	>800	>800	>800
TMPAL-12 Cl	160	160	80	160	>800
TMPAL-12 Br	20	80	40	80	650
TMPAL-10 Br	80	20	5	10	40

### Short-Time Killing

The short-time killing was investigated using the two most active gemini QAS (TMPAL-10 Br and TMPAL-12 Br) against *S. epidermidis* B374 (Table 2). It was shown that these two surfactants already exhibited a lethal effect against *S. epidermidis* cells after 5 min (0.15 % survival) and reached 100 % lethality after 60–120 min (Table 3). There were also some significant differences in the effect on cell survival between these two compounds. After 5-min contact TMPAL-10 Br is more lethal towards bacterial cells (*p* = 0.0408), however additional incubation up to 60 min showed greater impact of TMPAL-12 Br on the survival (*p* = 0.0065).

**Table 3:** The fraction of surviving *S. epidermidis* B374 cells after short-time exposure to alanine-derived gemini surfactants at MBC

Compound	Time of exposure [min]					
	0	5	15	30	60	120
TMPAL-10 Br [20 µM]	1.00	0.00155 ± 0.000212	0.0011 ± 0.0004242	0.00065 ± 0.000035	0.00021 ± 0.00009	0
TMPAL-12 Br [80 µM]	1.00	0.0165 ± 0.00495	0.001135 ± 0.000799	0.000355 ± 0.0001	0	0

Results represent the means ± SD of three independent experiments

### Adhesion

**Fig. 2:**
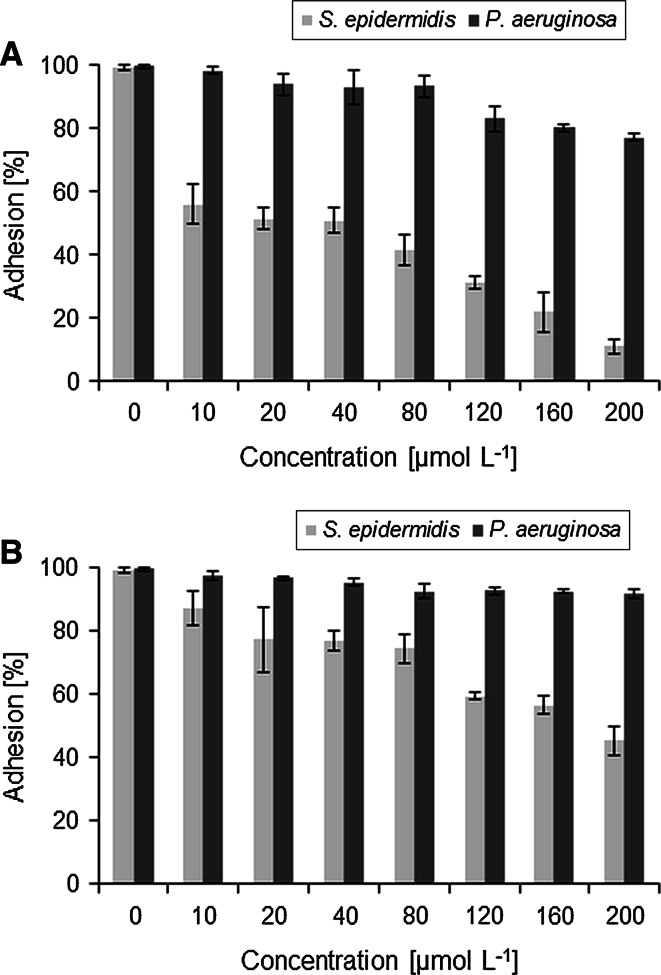
The inhibition of bacterial adhesion to the polystyrene by alanine-derived gemini surfactants: **a** TMEAL-12 Br; **b** TMPAL-12 Br. Results represent the means ± SD of three independent experiments

**Fig. 3:**
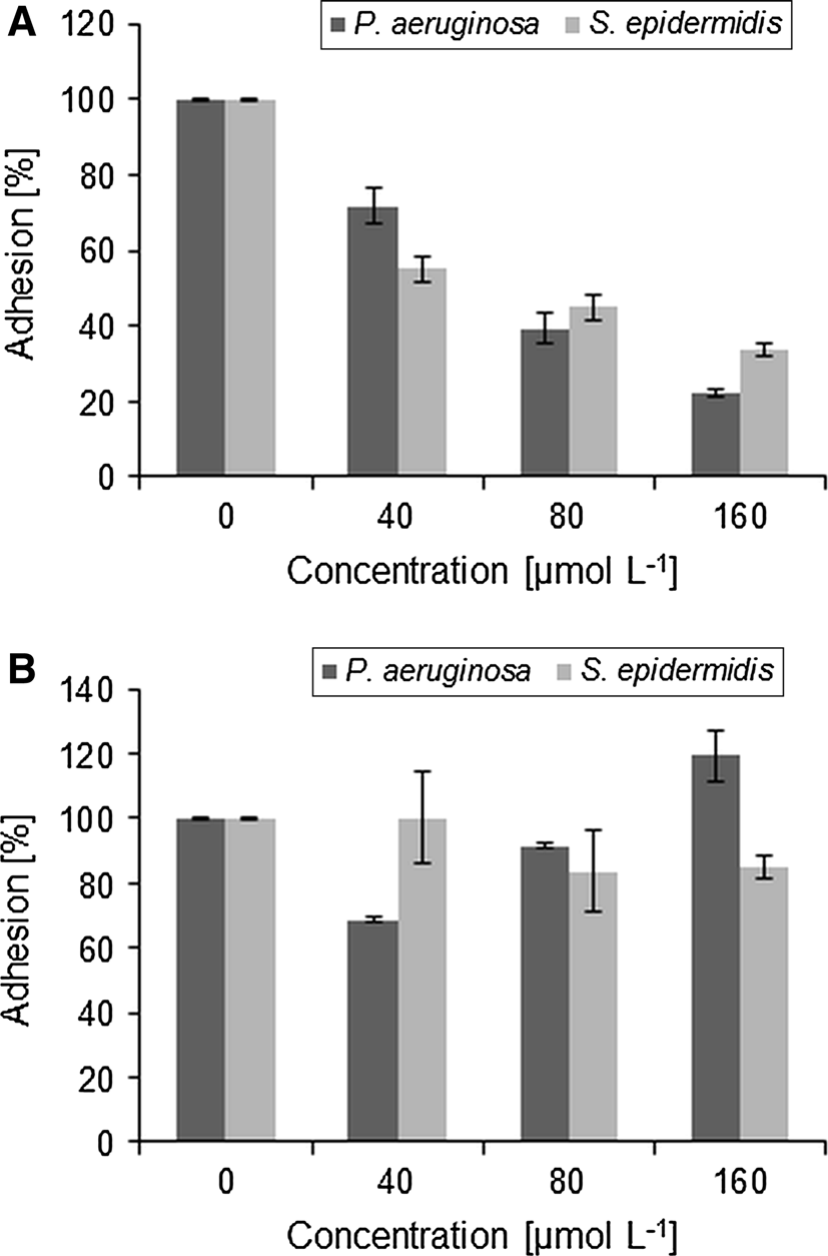
The inhibition of bacterial adhesion to the silicone surface by alanine-derived gemini surfactants: **a** TMEAL-12 Br; **b** TMPAL-12 Br. Results represent the means ± SD of three independent experiments

The adhesion of bacterial cells to the surface is the first stage of biofilm development. The gemini quaternary ammonium salts may be able to coat the surface and prevent cell adhesion. The research on adhesion inhibition showed that only the compounds with 12 carbon atoms within alkyl chains reduced *S. epidermidis* adhesion to a polystyrene plate. A greater inhibition was displayed by TMEAL-12 Br, which reduced *S. epidermidis* adhesion by about 50 % at a low concentration (20 µM), but significant reduction was observed already at 10 µM (*p* = 0.0035). On the other hand, *P. aeruginosa* adhesion to polystyrene was not inhibited to this extent by any of the compounds tested. However, TMEAL-12 Br showed a significant reduction in bacterial adhesion (by about 20 %) at 120 µM (*p* = 0.01) (see Fig. 2). TMEAL-12 Br was also effective in coating silicone catheters, since it significantly reduced the adhesion of both *S. epidermidis* (*p* = 0.017) and *P. aeruginosa* (*p* = 0.038), whereas TMPAL-12 Br did not show any significant anti-adhesive activity (Fig. 3).

### Biofilm Viability

**Fig. 4:**
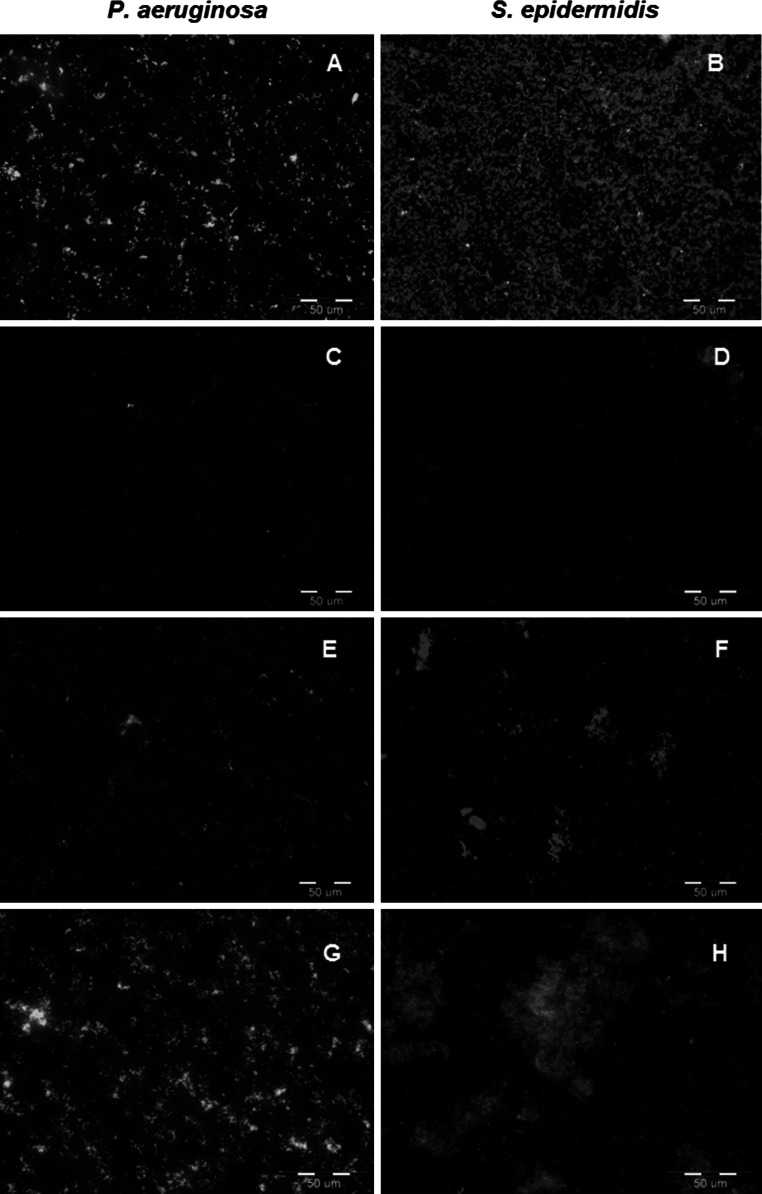
Syto9/PI staining of bacterial biofilms treated with 120 µmol L^−1^ alanine-derived gemini QAS. **a** and **b** untreated biofilms; **c** and **d** -TMPAL-10 Br; **e** and **f** TMPAL-12 Br; **g** and **h** TMEAL-12 Br. *Scale bar* 50 µm

Staining of bacterial biofilms with SYTO9/propidium iodide showed that *P. aeruginosa* PAO1 biofilm was eradicated by TMPAL-10 Br and TMPAL-12 Br. The amount of observed adherent cells was lower in comparison to the control, however the remaining biofilm was viable (green fluorescence) (Fig. 4a, c, e). *S. epidermidis* biofilm, on the other hand, was much more sensitive to gemini QAS. Both TMPAL-10 Br and TMPAL-12 Br caused a large drop in biofilm viability, manifested by the red fluorescence of propidium iodide (Fig. 4b, d, f). TMEAL-12 Br did not exhibit any significant effect on *P. aeruginosa* and *S. epidermidis* biofilms (Fig. 4g, h).

## Discussion

Gemini quaternary ammonium salts are a class of surfactants built of two monomeric QAS molecules linked by a spacer [21]. They exhibit stronger surface and biological activity in comparison to conventional QAS, which are widely used as drugs and disinfectants [22–24]. Due to the overuse of cleaning agents, there is a problem of increasing microbial cross-resistance, that could be overcome by the development of new antimicrobial compounds [1].

Gemini QAS show strong antibacterial and antifungal activity [25, 26]. Betaine- and alanine-derived gemini surfactants did not show cytotoxic effects towards yeast mitochondrial metabolism and they were not mutagenic [9, 27]. Previously investigated gemini QAS, having betaine-based, ester-type alkyl chain arrangements, inhibited *S. aureus* growth at low concentrations (10–80 µM). Chlorides with 10 and 12 carbon alkyl chains were also effective in eradicating *P. aeruginosa* [8]. Compared with betaine QAS surfactants, alanine derivatives with shorter spacer show weaker antibacterial activity both against Gram-positive and Gram-negative bacteria. On the other hand, elongation of the spacer increased biological activity to a higher level than in the case of betaine QAS gemini surfactants.

The strongest antibacterial effect was exhibited by bromides with longer spacers and alkyl chains of 10 or 12 carbons (TMPAL-10 Br and TMPAL-12 Br). These compounds inhibited growth of Gram-positive and Gram-negative strains at low concentrations and, more importantly, they were lethal to *S. epidermidis* after a short time of exposure. It has been shown previously that the biological activity of gemini surfactants depends on the spacer length. When the distance between alkyl chains was larger, the incorporation of surfactant into the plasma membrane of erythrocytes was easier and, in consequence, an increased level of cell disruption was observed [16].

The compounds with a shorter spacer (TMEAL-10 Br, TMEAL-12 Br) showed good antibacterial activity, but only against Gram-positive strains, since *P. aeruginosa* and *E. coli* exhibited tolerance to higher concentrations of these gemini QAS. A similar effect was observed for betaine QAS gemini surfactants [8]. The differences in gemini surfactant tolerance between Gram-positive and Gram-negative bacteria might be connected with the cell envelope structure. The presence of LPS molecules, outer membrane proteins or numerous efflux systems in *P. aeruginosa* and *E. coli* might be responsible for the resistance to gemini QAS [28, 29].

Many bacterial strains are able to live either as planktonic forms or in the biofilm structure. Biofilms are multicellular bacterial communities composed of cells surrounded by extracellular polymeric substances (EPS). EPS contains mostly polysaccharides, proteins and nucleic acids and protects the community from changes in environmental conditions. Bacterial biofilms are hard to eradicate due to the increased resistance to antimicrobial agents. The bacterial ability to form biofilms is often a cause of infections associated with medical devises (e.g., catheters) [14, 30]. The biofilm development starts with cell attachment to biotic or abiotic surfaces and this process involves many properties of the cell. Preventing bacterial adhesion is one of the strategies to stop biofilm formation and to counteract bacterial pathogenesis [31]. One of the modes of adhesion inhibition is changing the surface properties by anti-adhesive coatings. The examples include silver, heparin or sparfloxacin coatings of catheters or QAS-containing dental polymers [32–34]. It was previously shown that betaine QAS gemini surfactants with C12 and C14 carbon alkyl chains coat the polystyrene surface and reduce *S. epidermidis* adhesion and biofilm development [8]. Similarly, alanine-derived QAS gemini surfactants with 12 carbon alkyl chains exhibited anti-adhesive properties on the polystyrene surface at low concentrations, but only against *S. epidermidis*. Surprisingly, the coating of silicone catheters with TMEAL-12 Br inhibited cell adhesion of both *S. epidermidis* and *P. aeruginosa*. These results might suggest that this compound coated silicone more effectively than polystyrene and the amount of gemini QAS molecules deposited on the catheter is enough to block cell adhesion and reduce *P. aeruginosa* biofilm formation.

Mature bacterial biofilms exhibit increased tolerance to disinfectants and antibiotics. There are numerous determinants for the resistance, e.g., altered metabolism of adherent cells, overexpression of degrading enzymes, active efflux and poor penetration of biofilm structure by drugs [35]. Previously studied betaine-like gemini bromide with 12 carbon alkyl chains showed strong biofilm-dislodging properties against both *P. aeruginosa* and *S. epidermidis* [8]. Alanine derivatives with 10 and 12 carbon alkyl chains (TMPAL-10 Br and TMPAL-12 Br) reduced biofilm formation by both these species. What is more, the remaining adherent cells of *S. epidermidis* were killed after exposure to gemini QAS, since they failed to exclude propidium iodide. On the other hand, *P. aeruginosa* undislodged biofilm remained viable.

Although overall comparison of betaine- and alanine-derived gemini surfactants indicates that the latter exhibit weaker biological activity, the alanine bromides with longer spacer (TMPAL-*n* Br) have lower MIC and MBC and might in the future be considered for further studies towards application in medicine and industry.
